# Identification and classification of distinct surface markers of T regulatory cells

**DOI:** 10.3389/fimmu.2022.1055805

**Published:** 2023-01-19

**Authors:** Agnieszka S. Wegrzyn, Anna E. Kedzierska, Andrzej Obojski

**Affiliations:** ^1^ Łukasiewicz Research Network - PORT Polish Center for Technology Development, Bioengineering Group, Wroclaw, Poland; ^2^ Hirszfeld Institute of Immunology and Experimental Therapy, Polish Academy of Sciences, Wroclaw, Poland; ^3^ Department of Internal Medicine and Allergology, Wrocław Medical University, Wrocław, Poland

**Keywords:** regulatory T cells, conventional T cells, surface markers, Treg markers, activated T cells, phenotyping, flow cytometry

## Abstract

**Background:**

Regulatory T (Treg) cells have emerged as key players in the maintenance of immune homeostasis. Although significant progress has been made in recent years to define the Treg surface markers involved with or identifying their suppressive function, there remains much to be elucidated, and many questions persist. This study determined the expression of surface markers on human peripheral Treg cells and conventional T (Tconv) cells in a steady state and after activation to gain insight into their mechanism of action and more precisely characterize this regulatory population in humans.

**Methods:**

To screen Treg and Tconv cells, peripheral blood mononuclear cells (PBMCs) were isolated from volunteers, stained with a commercially available lyophilized antibody array comprising 371 surface antigens, and analyzed by flow cytometry. To compare Treg cells with activated Tconv cells, PBMCs were stimulated with PMA and further stained similar to freshly isolated cells.

**Results:**

Treg and Tconv cells were positive for 135 and 168 of the 371 antigens, respectively. Based on the frequency distribution, all of the most highly expressed markers identified were shared by both Treg and Tconv cells and participate in T cell activation, act as costimulatory and signaling molecules, or exhibit adhesion and migratory functions. Additionally, we identified several differences in marker expression between Treg and Tconv cells, with most found in the expression of co-stimulatory (ICOS, GITR, 4-1BB) and co-inhibitory (TIGIT, CTLA-4) molecules, as well as chemokine receptors (CXCR4, CXCR5, CCR4, CCR5, CCR7, CCR8, and CXCR7). Furthermore, post-activation expression of surface molecules identified molecules capable of discriminating Treg cells from activated Tconv cells (GITR, 4-1BB, TIGIT, CD120b, and CD39); however, almost all of these markers were also expressed in a small fraction of activated Tconv cells.

**Conclusions:**

These results offer insight into the biology of Tregs and contribute to their accurate identification and characterization in variety of immunological diseases as well as physiological processes

## 1 Introduction

Since the identification of cluster of differentiation (CD)4^+^CD25^+/hi^ cells as T regulatory (Treg) cells in humans in 2001 (1–3), they have remained the subject of intense research. In recent years, significant effort has been given to characterizing and studying this T cell population. Treg cells have broad functionality; participate in the development of tolerance to autoantigens, antigens of commensal microbiota, or food antigens; dampen the excessive immune response; regulate fetoplacental immunity; and play a key role in the homeostasis and regenerative processes of various tissues (4–8). Treg cells have been extensively studied for their involvement in the pathogenesis and therapy of various inflammatory diseases, such as autoimmune diseases, cancers, allergies, transplantation, and tissue engineering (8–10). Although the use of Treg cells for therapeutic purposes is attractive, such applications remain difficult in the absence of detailed knowledge of their biology.

To date, significant progress has been made in characterizing Treg cells and their role in health and disease; however, much remains to be elucidated. The heterogeneity of Treg cells and the lack of exclusive markers for clearly defining Treg subtypes make this area of study highly complex. To date, various surface molecules have been suggested as Treg markers [e.g., CD25, cytotoxic T lymphocyte antigen 4 (CTLA-4), glucocorticoid-induced tumor necrosis factor receptor (TNFR)-related protein (GITR), and inducible T cell co-stimulator (ICOS)]; however, none of these are exclusively expressed on Treg cells (11). The precise identification of Treg cells is challenging, because the expressed proteins are mostly shared by activated conventional effector T cells (Tconv).

Numerous recent studies indicate that Treg cells are not only homogeneous in phenotype but also equipped with various suppressive mechanisms. Tregs interact with several immune cells in a contact-dependent manner that results in the production of inhibitory cytokines, cytokine deprivation, cytolysis, apoptosis, and/or metabolic disruption (8, 12, 13). Despite progress in the field of Treg biology, a new basis for the reliable delineation of human Treg cells is still required. Updated knowledge will offer insight into their mechanism of action and precisely characterize this regulatory population in humans; therefore, in this study, we determined the expression of surface markers present on human peripheral Treg cells and Tconv cells in steady state and after activation.

To screen Treg and Tconv cells, peripheral blood mononuclear cells (PBMCs) were isolated from volunteers, stained with a commercially available lyophilized antibody array comprising 371 surface antigens, and analyzed by flow cytometry. To assess differences between activated Tconv and Treg cells, PBMCs were stimulated before staining.

The results shed light on the biology of Treg cells and update the current knowledge about their expression of surface molecules. Additionally, stratification of expressed surface molecules based on frequency allows for the prediction of markers responsible for cardinal functions of T cells, such as antigen recognition, adhesion, and migration or regulatory functions, with such knowledge potentially providing a better understanding of Treg cell dysfunction in disease. Although not focused on the identification of markers exclusively expressed on the surface of all Treg cells, this study identified markers highly expressed by the majority of Treg cells but not activated Tconv cells; thereby enabling delineation of these two subsets.

## 2 Methods

### 2.1 Blood samples

Peripheral blood were obtained from 7 volunteers (4 females and 3 males) aged 28 to 44 years. Volunteers were recruited from the staff of the Lukasiewicz Research Network, PORT Polish Center of Technology Development (Wroclaw, Poland). Before peripheral blood collection, a clinical interview was performed to exclude diseases that could affect the results of the study. All were non-smokers, non-obese, without diagnosed immune disease, and with no history of recent infection. Ethical permission for experiments with human blood was obtained from the Regional Ethical Review Board in Wroclaw, Poland (approval number: 593/2016), and informed consent was obtained from volunteers before blood collection.

### 2.2 Cell culture

Human PBMCs were isolated from peripheral venous blood using Ficoll-Hypaque (Sigma Aldrich, St. Louis, MO, USA) density gradient centrifugation. Freshly isolated PBMCs were kept overnight at 4°C with continuous mixing on a roller mixer in RPMI-1640 supplemented with 2 mM L-glutamine, 1% (v/v) nonessential amino acids and minimum essential vitamin solution, 1 mM sodium pyruvate, 100 U/mL penicillin, 100 mg/mL streptomycin, 50 mg/mL kanamycin, 50 mM β-mercaptoethanol, and 10% (v/v) heat-inactivated fetal calf serum (all from Gibco, Grand Island, NY, USA). For determination of expressed surface markers after activation, PBMCs were stimulated with 20 ng/mL phorbol 12-myristate 13-acetate (PMA) and 1 μg/mL ionomycin at 37°C and 5% CO_2_ for 2 h (all from Sigma-Aldrich).

### 2.3 Staining and flow cytometry analysis

To screen Treg cells, freshly isolated or stimulated PBMCs were stained with a commercially available lyophilized antibody array comprising 371 surface markers (BioLegend, San Diego, CA, USA) and co-stained with the following antibodies: anti-human CD25 Alexa Fluor 647 (clone BC96), anti-human CD127 PE/Cy7 (clone A019D5), anti-human CD4 PCP/Cy5.5 (clone RPA-T4), and anti-human CD14 APC/Cy7 (clone HCD14) to identify Treg cells (all from BioLegend). To identify Treg cells (to compare two methods of identification of Treg cells), the following antibodies were used: anti-human CD4 PCP/Cy5.5 (clone RPA-T4), anti-human CD25 BV421 (clone BC96), anti-human CD127 PE (clone A019D5), and anti-human FOXP3 Alexa Fluor 488 (clone 259D) (all from BioLegend). To assess selected surface-molecule expression in FOXP3^+^ cells, the following antibodies were used: anti-human CD8 APC/Cy7 (clone RPA–T8), anti-human CD14 APC/Cy7 (clone HCD14), anti-human CD16 APC/Cy7 (clone 3G8), anti-human CD19 APC/Cy7 (clone HIB19), anti-human CD56 APC/Cy7 (clone HCD56) for exclusion from cytotoxic T cells, monocytes, B-cells and natural killer cells (all from Biolegend). To characterize FOXP3^+^ cells, we used anti-human CD4 BV510 (clone OKT4), anti-human FOXP3 Alexa Fluor 647 (clone 259D, Biolegend), anti-human CD95 FITC (clone DX2), anti-human CD120b BV421 (clone hTNFR-M1), anti-human T cell immunoreceptor with Ig and ITIM domains (TIGIT) BV421 (clone 741182), anti-human CD39 BB515 (clone TU66), anti-human CD137 PE (clone 4B4-1), and anti-human GITR BB700 (clone V27-580) (all from BD Biosciences, Franklin Lakes, NJ, USA). PBMCs for this experiment were stained using two separate antibody panels. In all experiments, PBMCs were stained with a fixable viability dye (fixable viability dye eFluor780; eBioscience, Thermo Fisher Scientific, Waltham, MA, USA) to exclude dead cells before staining with antibodies. Following viability and surface staining, PBMCs were fixated and permeabilised with True-Nuclear™ Transcription Factor Buffer Set (Biolegend) and further stained with FOXP3 according to manufacturer’s protocol. The cells were analyzed by flow cytometry (BD FACS Canto II or BD LSR Fortessa; BD Biosciences), in array analysis, 10000 events in live CD4+ cells gate were acquired, and for the remaining experiments, 50000 events were recorded in live CD4+ cells gate. To standardize the flow cytometric readouts across time, application settings were applied in each experiment. Data were analyzed using FlowJo software (FlowJo LLC, Ashland, OR, USA).

### 2.4 Statistical analysis

For statistical analysis, we applied the Mann-Whitney or Kruskal–Wallis test, followed by the Dunn *post hoc* test for multiple comparisons, with a P < 0.05 considered significant. Correlation analysis was performed by Spearman or Pearson test. All statistical analyses were performed using GraphPad Prism software (GraphPad Software, Inc., La Jolla, Calif, USA).

## 3 Results

### 3.1 The majority of surface markers are expressed by both Treg and Tconv cells

**Figure 1:**
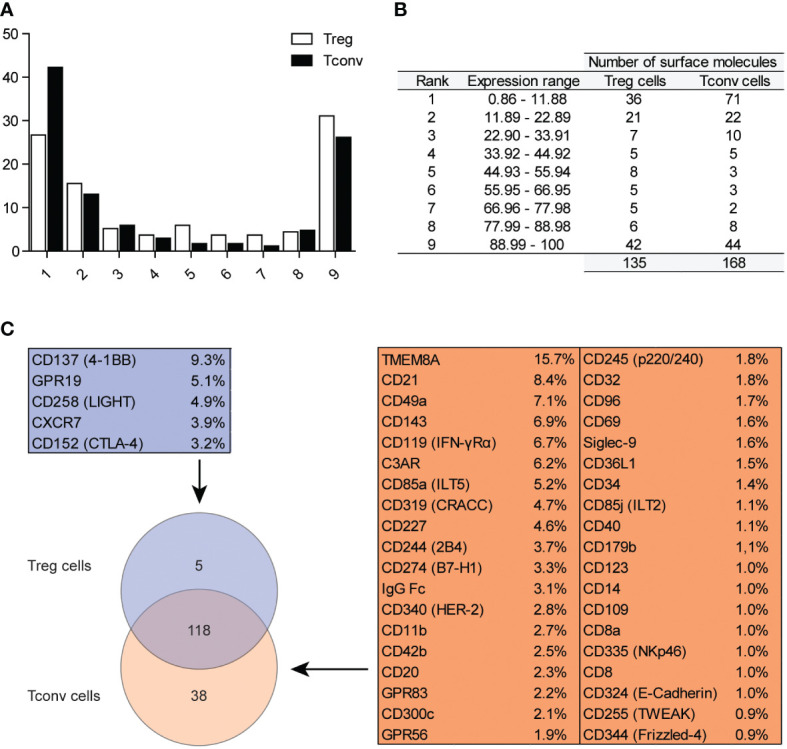
Classification of surface markers on Tconv and Treg cells. **(A)** Graphical presentation of frequency distribution of surface markers on Treg (n=5) and Tconv cells (n=5). **(B)** Tabular presentation of classification of surface markers based on their expression on Treg (n=5) and Tconv (n=5). **(C)** Venn diagram showing the identification of surface markers exclusively expressed on Treg or Tconv cells. Data are presented as mean (n=5, for both Treg and Tconv).

To investigate the expression of surface molecules on Treg and Tconv cells, we used an antibody matrix array, which requires a large amount of starting material for staining. Generally, long sample-preparation procedures (e.g., identification of intracellular antigens) are associated with reduced numbers of cells in a sample owing to the numerous washing steps required during the staining procedure. Therefore, we assessed the efficiency of the two different methods used for Treg cell identification (**Supplementary Figure 1A**). Treg cells were identified simultaneously as CD25^+^FOXP3^+^ (requiring intracellular staining) or CD25^+^CD127^lo/−^ (based only on surface-marker expression). The correlation of Treg cell frequencies achieved by different identification methods (**Supplementary Figure 1B**) revealed comparable results; therefore, surface-marker screening for Treg cells targeted CD25^+^CD127^lo/−^ subsets of CD4^+^CD14^−^ cells (**Supplementary Figure 1C**). The gate for positive cells for respective antigens was established based on the fluorescence minus one control (**Supplementary Figure 1C**). The surface markers were divided into nine classes according to the frequency of their respective antigens (**Figures 1A, B**), with the majority of surface markers positioned in the first, second, and ninth groups. The results showed that Treg and Tconv cells were positive for 135 and 168 among the 371 checked antigens, respectively (**Figure 1B**), with a total of 118 antigens expressed by both Treg and Tconv cells. Only five antigens were exclusively expressed by Treg cells, and 38 were expressed by Tconv cells (**Figure 1C**); however, <10% of Treg cells and 16% of Tconv cells exhibited expression of these markers. The differences in the numbers of such antigens were the result of the assumed population size being reliable (0.89% positive for Tconv and 2.99% for Treg cells according to a Poisson distribution. The percentages of cells positive for all surface markers available in the antibody matrix array are summarized in **Supplementary Figure 2**.

### 3.2 Highly expressed markers are associated with cardinal T cells functions

**Figure 2:**
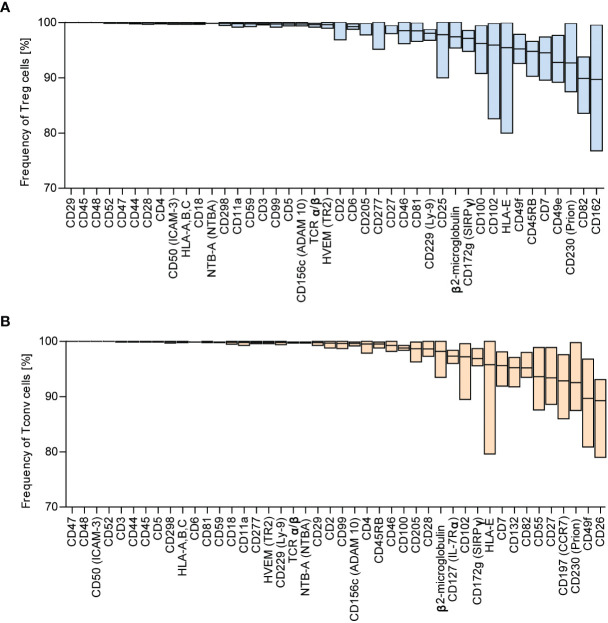
Surface markers are highly expressed across Tconv and Treg cells. Based on frequency distribution analyses, the highly expressed surface markers were identified (**Figure 1B**). The percent positive cells for the given marker were in the range of 88.99 – 100%. **(A)** Treg and **(B)** Tconv cells. Data are presented as floating bars (min to max, the line indicates mean) and show the percentage of Treg or Tconv cells positive for distinct surface markers (n=5, for both Treg and Tconv).

We identified the most highly expressed markers based on the frequency distribution. The numbers of identified antigens were similar in both groups: 42 in Treg and 44 in Tconv cells (**Figure 1B**), with most of the identified surface molecules expressed by almost all Treg and Tconv cells (**Figures 2A, B**). The most highly expressed surface markers exhibited relatively low inter-donor variability; however, analysis of median fluorescence intensity revealed high heterogeneity between markers that was not proportional to the percentage of positive cells (**Supplementary Figures 3A, B**). Identified markers are engaged in cardinal T cell functions and could be stratified into the following categories according to function: 1) T cell activation *via* the T cell receptor (TCR) complex (CD3, CD4, CD45, TCRα/β, CD277); 2) T cell-activation molecules (CD25, CD127, CD100, CD230, NTB-A, CD229); 3) co-stimulatory molecules (CD6, CD7, CD28, CD45RB, CD81, CD82); 4) signaling molecules (CD5, CD59); 5) adhesion and migratory molecules (CD2, CD11a, CD18, CD29, CD44, CD47, CD48, CD49f, CD49e, CD50, CD99, CD102, CD162); and 6) molecules involved in antigen presentation [human leukocyte antigen (HLA)-A/B/C, HAL-E, β2-microglobulin]. Other molecules that are broadly expressed on Treg and Tconv cells exhibit enzymatic activity (CD156c, CD298), are involved in antigen uptake (CD277), belong to the tumor necrosis factor receptor (TNFR) superfamily (herpes virus entry mediator; HVEM), protect autologous cells from complement attack, and have an unknown function in T cells (CD52). Given the high percentage of T lymphocytes expressing the identified surface molecules, which were also expressed in both subpopulations (Treg and Tconv cells), this suggested that the expression of such molecules is common in T cells. However, these data do not qualify these surface molecules as lineage-specific molecules. Comparison of surface expression on leukocytes other than T cells is required for a comprehensive review.

### 3.3 Treg cells exhibit higher expression of co-stimulatory and co-inhibitory molecules and chemokine receptors relative to Tconv cells

**Figure 3:**
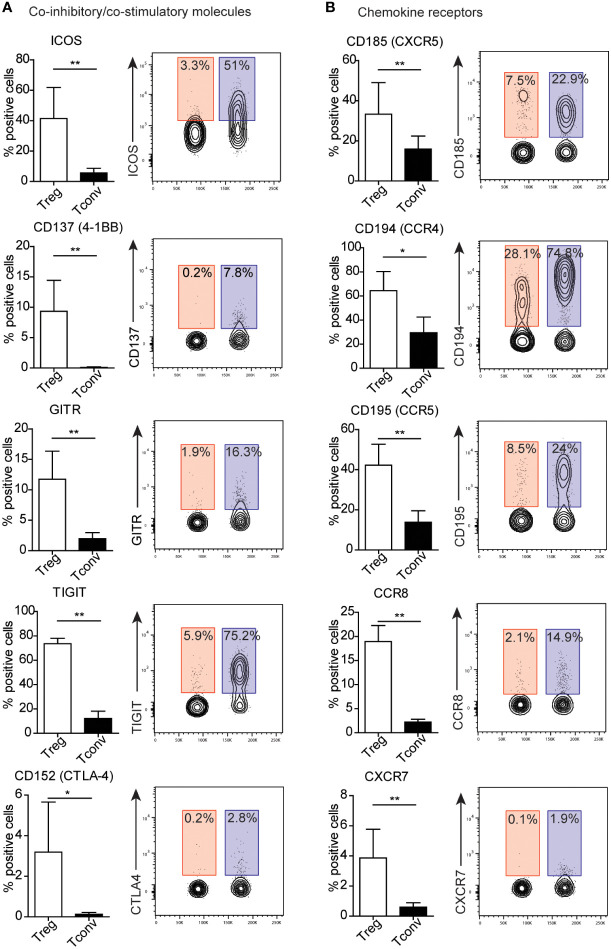
Treg cells exhibit distinct patterns of expression of co-inhibitory and co-stimulatory molecules and chemokine receptors. **(A)** Co-inhibitory and co-stimulatory molecules and **(B)** chemokine receptors, Numbers adjacent to gates on contour plots indicate the percent gated cells as positive for the given surface marker (blue – Treg, red – Tconv cells). To compare Treg and Tconv cells, plots were made by the concatenation function by FlowJo. Data are presented as bars (n=5, for both Treg and Tconv). All data represent the mean ± SD. *P ≤ 0.05 and **P ≤ 0.01 (Mann Whitney test **(A, B)**.

Because we identified no surface molecules exclusively expressed on Treg or Tconv cells, we identified alterations in expression between both populations (**Figure 3** and **Supplementary Figures 4**–**5**). To simplify the analysis of differences in expression, surface markers were stratified into groups according to their function. We identified the majority of differences among the co-inhibitory and co-stimulatory molecules and chemokine receptors (**Figures 3A, B**; and **Supplementary Figures 5A, B**). Treg cells exhibit higher expression of regulatory proteins, such as ICOS, CD137 (4-1BB), GITR, TIGIT, and CD152 (CTLA-4). A prominent difference in the percentage of Tconv versus Treg cells involved presentation of TIGIT and ICOS (**Figure 3A**). Additionally, Tconv cells exhibited a higher frequency of CD226 (a member of the immunoglobulin superfamily) and B and T lymphocyte attenuator (BTLA) than Treg cells (**Supplementary Figure 5A**). Moreover, Treg and Tconv cells express a broad range of chemokine receptors (**Supplementary Figure 2**), with Tregs demonstrating higher expression of chemokine receptors, such as C–X–C motif chemokine receptor (CXCR)5 (CD185), CC motif chemokine receptor (CCR)4 (CD194), CCR5 (CD195), CCR8 (CD198), and CXCR7 (**Figure 3B**), relative to Tconv cells, whereas Tconv cells showed higher expression of CXCR4 (CD184) and CCR7 (CD197) than Treg cells (**Supplementary Figure 5B**). However, both CXCR4 and CCR7 are broadly expressed by Tregs. Notably, the frequency of cells positive for chemokine receptors showed high inter-donor variability and might be dependent on the T cell differentiation, activation and polarization (14–17). Another difference in the percentages of Treg and Tconv cells involved the expression of the adhesion and migratory molecules CD166, sialyl-Lewis X, CD49d, and integrin b7 (**Supplementary Figures 4A** and **5C**). Similar to chemokine receptors, we also observed high inter-donor variability in these cases. Furthermore, Treg and Tconv cells differentially expressed cytokine receptors (**Supplementary Figures 4B** and **5D**), with the most noticeable difference in the percentage of cells presenting interleukin-2 receptor α chain (IL-2Rα, CD25) and IL7Rα (CD127), respectively. Both Treg and Tconv cells expressed IL6Rβ (CD130); however, a statistically higher percentage of these cells was observed in the Tconv subset, whereas most Treg cells were positive for TNFR2 (CD120b). One difference in the frequency of surface molecules involved the presence of the TCR complex (**Supplementary Figure 4D**). Treg cells showed a higher frequency of expression of the membrane protein tyrosine phosphatase CD148 and the MHC class 2 molecule HLA-DR, with most also positive for CD45RO, a marker of human memory T cells, and the activation marker CD71 (**Supplementary Figure 4E**). By contrast, Tconv cells showed a higher percentage of cells expressing complement regulation protein (CD55) and membrane transporter (CD243) relative to Tregs (**Supplementary Figures 5E, F**). We observed a further difference in the presence of proteins showing enzymatic activity within the subset populations, with a higher prevalence of CD39 found among Tregs and CD26 among Tconv cells, although ~60% of Treg cells presented CD26 expression (**Supplementary Figures 4C** and **Supplementary Figure 5G**).

### 3.4 Impact of activation on the expression surface molecules on Treg and Tconv cells

The identification of a specific marker for Treg lymphocytes is hampered by the increased expression of Treg lymphocyte markers in activated lymphocytes (11, 18). Therefore, we evaluated the expression of these markers following PBMC activation with PMA. PMA activates protein kinase C without TCR engagement, and cell activation is observed even after 30 min of exposure (19). Here, we stimulated cells with PMA for 2 h and observed elevated expression of early activation markers (CD69 and CD40L) in both Treg and Tconv cells (**Supplementary Figure 2**). A summary of the expression of all available surface markers in the antibody array after activation is shown in **Supplementary Figure 2**.

**Figure 4:**
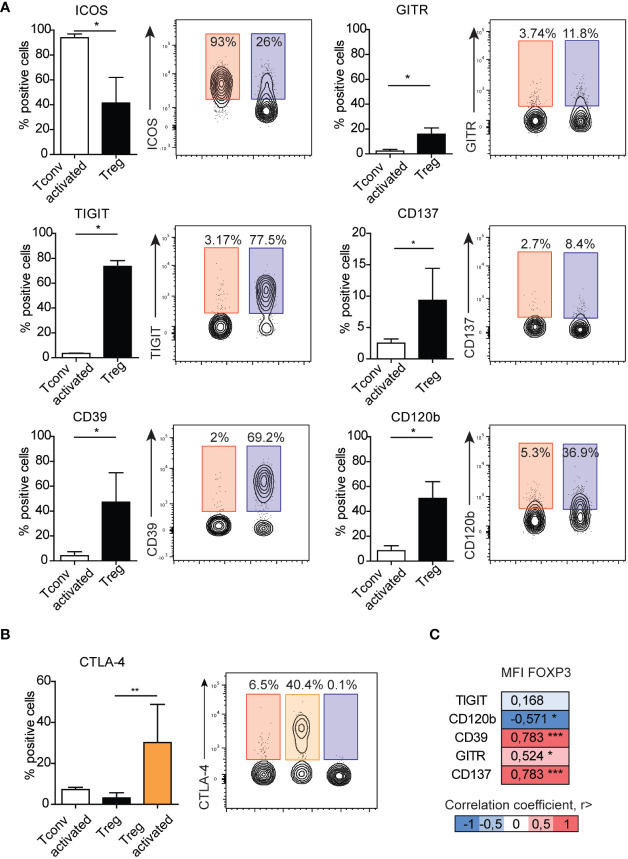
Surface expression of GITR, TIGIT, CD137, CD39, or CD120b distinguish activated Tconv from Treg cells. **(A)** The impact of activation on the expression of surface markers on Tconv (n=3) cells and comparison with those on Treg cells (n=5). **(B)** The impact of activation on the expression of CTAL-4 on Tconv (n=5) and Treg (n=5, n=3 activated Treg) cells. **(C)** Correlation between the median fluorescence intensity of FOXP3 and Treg-related surface markers. Numbers adjacent to gates on contour plots indicate percent gated cells as positive for the given surface marker (blue – Treg, red – activated Tconv, orange – activated Treg cells). To compare Treg and Tconv cells, plots were made by the concatenation function by FlowJo. Data represent the mean ± SD. *P ≤ 0.05, **P ≤ 0.01 and ***P≤0.001 (Mann Whitney test **(A)**, Kruskal-Wallis test **(B)**, Spearman correlation **(C)**).

Because we observed a majority of differences between Treg and Tconv cells between the presentation of co-stimulatory and co-inhibitory molecules, they were selected for further analysis after activation. We found that ICOS expression increased in both activated Tconv and Treg cells (**Supplementary Figure 2**), with expression on activated Tconv cells significantly higher than that on Treg cells (**Figure 4A**) and suggesting it as a poor marker for extracting Treg subsets. In the Treg population, the frequency of GITR^+^ and CD137^+^ cells was higher than that of activated Tconv cells, although the percentage of these cells was only ~10%, suggesting that activation does not influence the frequency of CD137^+^ Tregs. Moreover, we found that the percentage of GITR^+^ cells decreased in the Treg population and was comparable to that among Tconv and activated Tconv cells (**Supplementary Figure 2**). The most noticeable difference in the frequency of activation-related surface markers between Treg and Tconv cells was in TIGIT presentation, with a small percentage of Tconv and activated Tconv cells showed TIGIT expression. Whereas Treg cell activation resulted in reduced TIGIT expression (**Figure 4A** and **Supplementary Figure 2**). Activation of Tconv cells did not affect CD39 or CD120b expression; however, the percentage of CD39^+^ cells increased in the Treg population after stimulation, whereas CD120b expression in activated Treg cells was reduced (**Figure 4A** and **Supplementary Figure 2**). Additionally, both activated Treg and Tconv cells demonstrated comparable expression of CTLA-4 (**Figure 4B**), whereas activated Treg cells exhibited a higher percentage of CTLA-4^+^ relative to unstimulated Tregs.

To date, all observations have been made on Treg cells and identified based on the expression of surface markers. Therefore, we focused on Treg marker expression relative to levels of FOXP3, the master transcription factor in Treg cells. Evaluation of surface markers in freshly isolated PBMCs without stimulation revealed that the frequencies of CD120b, CD39, GITR, and CD137 correlated with the median fluorescence intensity of FOXP3 (**Figure 4C**). We then analyzed the distribution of CD120b, TIGIT, CD39, CD137, and GITR presentation on FOXP3^+^ cells using t-distributed stochastic neighbor embedding (tSNE) (**Supplementary Figure 7A**). Given the limited availability of antibodies conjugated to different fluorochromes, which would allow for staining in a single panel, we evaluated the FOXP3^+^ population in two separate panels. tSNE analysis revealed that CD120b and TIGIT are widely expressed in FOXP3^+^ cells, and that their expression overlapped with that of CD95, a marker of Treg cell differentiation. Additionally, CD39, CD137, and GITR expression was restricted to a small subset of FOXP3^+^ cells. These observations suggested that CD120b and TIGIT might be classified as general Treg cell markers, whereas CD39, CD137, and GITR are related to a functional subset of Treg cells.

## 4 Discussion

A growing body of evidence indicates that Tregs are key players in controlling autoimmunity, allergic reactions, responses to tissue transplants, tumors, and infections (4–7, 20), with numerous studies showing that Tregs use diverse suppressive mechanisms to control immune responses (21, 22). Therefore, Treg cells differentiate into multifunctional cells that exhibit a broad spectrum of mechanisms that regulate the immune response. Despite previous efforts, Treg cells remain poorly characterized. Therefore, the identification of markers exclusively expressed on Treg cells or improved characterization of the Treg phenotype might aid in further development of biological therapies for inflammatory diseases.

This study presents unique insight into the surface-protein-expression profiles of human T cells and provides detailed information regarding the changes in expression after activation. The activation profile of T cells is applicable at least in Treg–cell based therapies for autoimmune diseases or cancer. The expanded *in vitro* Treg cells found application in the treatment of autoimmune diseases and prevention of GVHD or graft rejection (23–25). It has been shown that naive Treg cells represent high proliferative capacity (24, 26). Therefore, our results may contribute to the improvement of isolation Tregs with high proliferative potential and may reduce the risk of contamination with effector T cells. On the other hand, highly activated and terminally differentiated Treg cells infiltrate tumor tissue (27–30). Blocking by monoclonal antibodies highly activated and suppressive Treg cells might be good targets in anticancer therapy. Therefore, these findings serve as a useful tool to enhance the understanding of T cell function. Additionally, this knowledge promotes the development of new therapeutic interventions for controlling of a broad range of inflammatory disorders.

Although we specifically focused on several molecules that play a role in Treg activity, we were unable to identify selectively expressed surface markers on Tregs. One limitation of this study is the limited number of markers available in commercial antibody arrays comprising previously identified CD molecules. Despite this limitation, we were able to identify surface markers showing high expression on Treg cells, allowing for improved stratification between activated Tconv and Treg cells.

Highly expressed markers (>88.9% of positive cells) were shared by both Tconv and Treg cells, with the most abundant molecules expressed by the majority of lymphocytes being those involved in cell–cell adhesion or adhesion to the extracellular matrix. The majority of these molecules displayed elevated expression that remained stable after stimulation. We observed reduced expression for CD205, CD230, HLA-E, HVEM, and β2-microglobulin in both Treg and Tconv cells, and CD49f and CD229 levels were reduced in Tconv and Treg cells, respectively. All of these molecules are involved in processes that include T cell activation, co-stimulation, signaling, and adhesion and play a critical role in antigen recognition and development of the immune response. Defects in their expression are likely related to immune deficiencies manifested by opportunistic infections, autoimmunity, and trigger malignancies. Previous studies reported that defective expression of integrins CD11a/CD18 lead to a failure in leukocyte recruitment to infection sites (31, 32). Additionally, mutations in CD3 and CD45 result in defective TCR signaling (33), and deficiencies in CD55 and CD50 expression trigger excessive destruction of red cells and leukocytes from uncontrolled complement-mediated lysis (34). These findings support the identification of highly expression surface markers on Treg and Tconv cells as helpful in characterizing immunodeficiencies and autoimmune disease. However, additional research is required to confirm the function of the identified markers in T cell biology and their potential utility for disease diagnosis and/or as therapeutic targets in several immune diseases.

Despite the lack of success in selecting Treg-specific markers, several differences in the expression of many markers between Treg and Tconv cells have been identified. The most prominent differences were found in the expression of co-stimulatory (ICOS, GITR, and 4-1BB) and co-inhibitory (TIGIT and CTLA-4) molecules, as well as chemokine receptors (CXCR4, CXCR5, CCR4, CCR5, CCR7, CCR8, and CXCR7). Chemokines and chemokine receptor interactions are crucial for lymphoid development, homing to a range of tissues or sites of inflammation, and immunological regulation (35, 36). It was shown here that Treg cells exhibit a different pattern of expression of chemokine receptors compared to Tconv cells. This observation suggests a distinct migratory behavior of Treg and Tconv cells. It has already been documented that Treg cells undergo changes in trafficking receptors, depending on their activation and differentiation stages. Naïve Treg cells mainly express lymphoid tissue-homing receptors, such as CCR7 and CXCR4 (16). On the other hand, in secondary lymphoid organs, the expression of CCR7 and CXCR4 is diminished whereas the expression of CCR2, CCR4, CCR5, CCR6, CXCR3, CXCR5 and CXCR6 is sharply upregulated (16). As have been shown in the present study, majority blood Tconv and Treg cells are positive for CCR7 and CXCR4. It was expected that represents a naïve phenotype. In addition, Treg cells predominantly express non-lymphoid tissue homing receptors, such as CCR4, CCR5, CCR6, and CCR8, compared to Tconv cells. Based on this observation, Tregs are expected to be recruited to tissues to regulate inflammatory processes.

Analysis of the expression of surface molecules after activation allowed identification of those associated with differentiation of Treg cells from activated Tconv cells, including GITR, 4-1BB, TIGIT, CD120b, and CD39. However, almost all of these markers were also present in a small (<10%) fraction of activated Tconv cells. GITR and 4-1BB are members of the TNFR superfamily that exhibit co-stimulatory functions and are expressed on activated T cells (37). The roles of GITR and 4-1BB in Treg biology remain unclear and controversial. Stimulation *via* GITR reportedly attenuates the suppressive activity of Treg cells, whereas GITR-related signaling co-stimulates the proliferation of Tconv cells and allows their escape from suppression (38, 39). Similarly, 4-1BB enhances the proliferation of Tconv cells; however, 4-1BB^+^ Tregs are assumed to be more suppressive than 4-1BB^−^ Tregs. Moreover, previous reports suggest that Tregs present in the tumor microenvironment express high amounts of 4-1BB (40) and that an increased frequency of 4-1BB^+^ Tregs is associated with poor prognosis in lung adenocarcinoma patients (41). To date, most studies have focused on the functions of GITR and 4-1BB in cancer. Because the induction of both GITR and 4-1BB expression by Treg cells follows TCR activation, they might play a critical role in allergy and infection responses. Furthermore, we found that GITR and 4-1BB expression was positively correlated with the median intensity of FOXP3 expression, suggesting the possibility that GITR^+^ and 4-1BB^+^ Tregs are highly suppressive subsets.

We showed that TIGIT (a member of the Ig superfamily) and CD120b (TNFR2; a member of the TNFR superfamily) are broadly expressed by Treg cells but not in activated Tconv cells. tSNE analysis revealed that all Treg cells expressed CD120b, and that TIGIT expression overlapped with that of CD95. CD95^+^ Treg cells are terminally differentiated subpopulations of Tregs (11), which might suggest that TIGIT^+^ Treg cells exhibit effector or terminal effector phenotypes. TIGIT has been extensively studied in the context of cancer immunotherapy (42, 43). Additionally, a mouse model revealed that TIGIT can directly suppress T cell responses independent of antigen-presenting cells, and that loss of TIGIT in mice leads to increased susceptibility to autoimmunity (44). Another study demonstrated that TIGIT^+^ Treg cells specifically inhibit proinflammatory T helper (Th)1 and Th17 cells but not Th2 cell responses (45). Moreover, a previous study demonstrated that TIGIT enhances Th2 immunity in mice with experimental allergic diseases (46). Thus, TIGIT might also be useful as a therapeutic target in allergic diseases.

The expression of CD120b (TNFR2) is reportedly restricted to lymphocytes, especially for a subset of Tregs with maximal suppressive capacity. CD120b can bind both membrane-bound TNF and its soluble form, and interestingly, Treg cells can shed large amounts of CD120b from their surface. Therefore, this suggests that CD120b can inhibit the activity of TNF-α, an essential cytokine that drives inflammation (47). Additionally, the interaction between TNF-α and TNFR2 critically affects the activation, expansion, and phenotypic stability of Treg cells (48). Moreover, studies on human and murine cancers reveal that highly suppressive TNFR2^+^ Tregs are involved in cancer immune evasion (49, 50).

In contrast to CD120b and TIGIT, we found that CD39 was present in one region of the tSNE plot. We speculate that CD39^+^ Tregs are a distinct subset responsible for the removal of extracellular adenosine triphosphate (ATP), which exerts proinflammatory effects and elimination of which might exert anti-inflammatory effects. CD39 exhibits enzymatic activity capable of cleaving ATP to adenosine monophosphate, which can be further cleaved by CD73 to adenosine (51). CD39^+^ Treg cells are thought to be highly active, suppressive, and capable of secreting IL-10 (52). Additionally, animal models of allergic airway inflammation demonstrated that CD39^+^ Treg cells limit inflammation by regulating extracellular ATP and/or adenosine levels (53). Therefore, extracellular ATP might represent an underlying aspect of airway inflammation in asthma. Thus, its role in limiting inflammation might render CD39 an attractive therapeutic target.

In conclusion, these data underscore the high degree of Treg cell heterogeneity. However, the question remains whether Treg cells comprise many functionally distinct subsets or whether they can employ a broad spectrum of modes of action. These findings provide a large amount of data concerning the molecules capable of mediating Treg cell activity; however, their individual or collective importance remains to be established. Furthermore, the identification of molecules expressed by the majority of Tregs is useful for the separation of highly purified Tregs. Therefore, our findings promote the further development of Treg-specific therapeutic applications, as well as functional analysis of Treg cells in human diseases.

## Data availability statement

The original contributions presented in the study are included in the article/**Supplementary Material**. Further inquiries can be directed to the corresponding author.

## Ethics statement

The studies involving human participants were reviewed and approved by Regional Ethical Review Board in Wroclaw, Poland (approval number: 593/2016). The patients/participants provided their written informed consent to participate in this study.

## Author contributions

AW designed the study, conducted flow cytometry experiments, analyze data and wrote the manuscript, AK conducted flow cytometry experiments and wrote the manuscript, AO supervised the study and wrote the manuscript. All authors contributed to the article and approved the submitted version.

## Supplementary material

Supplementary Figure 1 Identification of Treg and Tconv cells. A. Representative dot plots for identification of Treg cells based on the expression of CD25 and FOXP3 or CD25 and CD127. B. Correlation between the frequencies of Treg cell subsets obtained by two different identification methods. Each symbol represents one donor (n=33). C. Representative dot plots and the gating strategy used to evaluate the expression of surface markers on Treg and Tconv cells (Tregs were identified as CD25^+^CD127^lo/−^ cells among a CD4^+^CD14^−^ population), followed by assessment of the expression of distinct markers between subsets.

Supplementary Figure 2 Overview of the frequency of distinct surface markers. A. Frequencies of individual surface markers on Treg and Tconv cells with or without activation. Data present the mean (Treg and Tconv cells without activation n=5, with activation n=3) and are presented as a heat map for better identification of positive cells (blue – low expression, red – high expression).

Supplementary Figure 3 Distribution of median fluorescence intensity. **(A)** Distribution of the median fluorescence intensity (MFI) of highly expressed markers on A. Treg (n=5) and **(B)** Tconv cells (n=5). Data are presented as floating bars (min to max, the line indicates mean).

Supplementary Figure 4 Identification of surface markers expressed mainly by Treg cells. **(A)** Adhesion or migratory molecules, **(B)** cytokine receptors, **(C)** markers with enzymatic activity, **(D)** molecules associated with the TCR complex, **(E)** activation markers, and **(F)** representative contour plots. Numbers adjacent to gates indicate the percent gated cells as positive for the given surface marker (blue – Treg, red – Tconv cells). To compare Treg and Tconv cells, plots were made by the concatenation function by FlowJo. Data are presented as bars (n=5, for both Treg and Tconv) and represent the mean ± SD. *P ≤ 0.05 and **P ≤ 0.01 (Mann Whitney test **(A–E)**).

Supplementary Figure 5 Identification of surface markers expressed mainly by Tconv cells. **(A)** Co-inhibitory and co-stimulatory molecules, **(B)** chemokine receptors, **(C)** adhesion or migratory molecules, **(D)** cytokine receptors, **(E)** complement regulatory proteins, **(F)** membrane transporters, and G. markers with enzymatic activity. Data are presented as bars (n=5, for both Treg and Tconv cells) and represent the mean ± SD. *P ≤ 0.05 and **P ≤ 0.01 (Mann Whitney test **(A–E)**).

Supplementary Figure 6 Representative contour plots. Contour plots showing the difference in expression surface markers shown in **Supplementary Figure 5**. Numbers adjacent to gates indicate the percent gated cells as positive for the given surface marker (blue – Treg, red – Tconv cells). To compare Treg and Tconv cells, plots were made by the concatenation function by FlowJo.

Supplementary Figure 7 Distribution of selected surface markers on FOXP3^+^ T cells. **(A)** tSNE analysis (n = 10/replicate) and the resulting plots and distribution of CD95, CD120b, TIGIT, CD39, CD137, and GITR on FOXP3^+^ T cells.
