# Supplementary figures and images for: Identification and classification of distinct surface markers of T regulatory cells
Source: Front Immunol. 2023 Jan 19;13:1055805. doi: 10.3389/fimmu.2022.1055805 (PMC9892051; doi:10.3389/fimmu.2022.1055805)

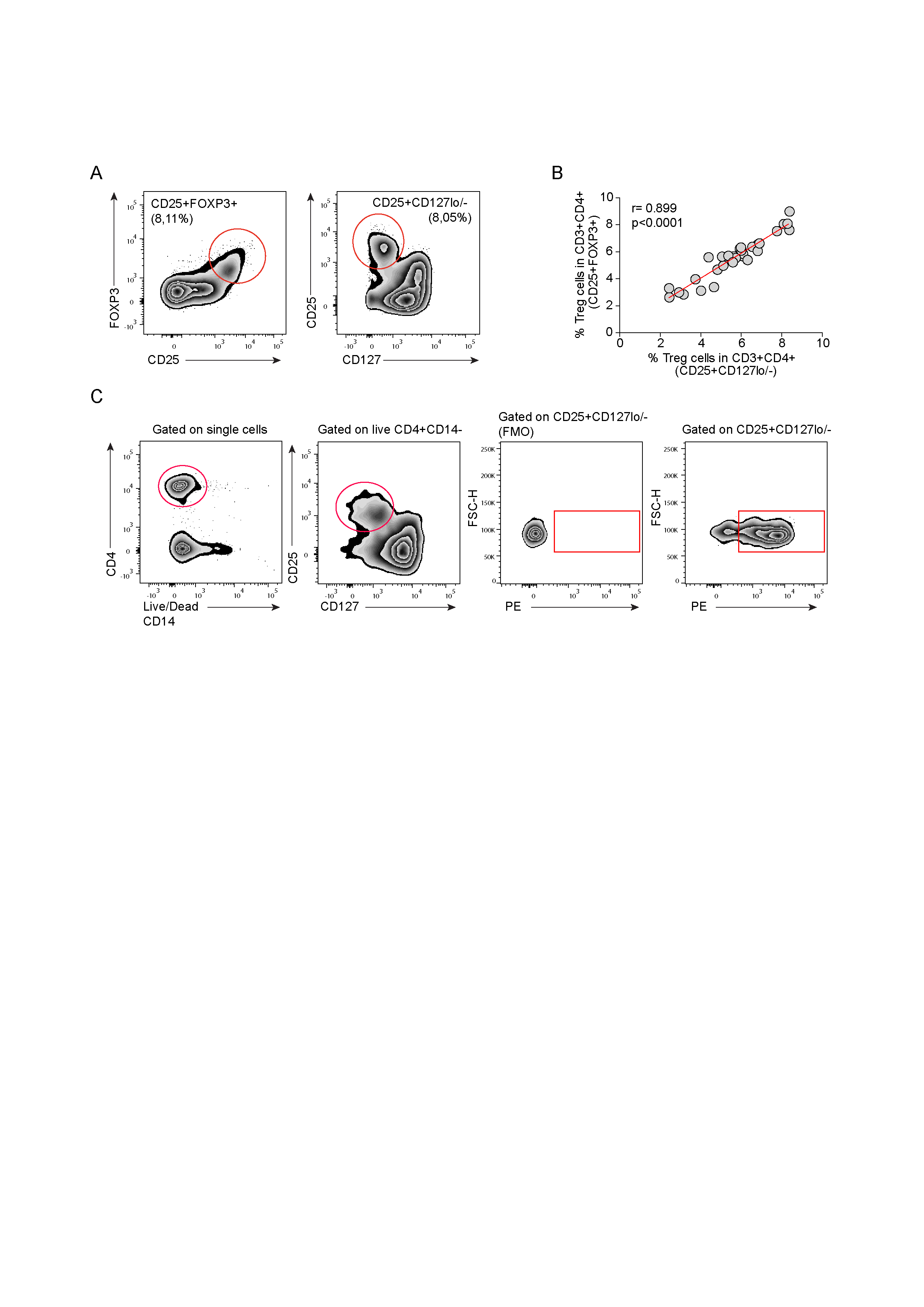

Supplement: Supplementary Figure 1 — Identification of Treg and Tconv cells. A. Representative dot plots for identification of Treg cells based on the expression of CD25 and FOXP3 or CD25 and CD127. B. Correlation between the frequencies of Treg cell subsets obtained by two different identification methods. Each symbol represents one donor (n=33). C. Representative dot plots and the gating strategy used to evaluate the expression of surface markers on Treg and Tconv cells (Tregs were identified as CD25+CD127lo/− cells among a CD4+CD14− population), followed by assessment of the expression of distinct markers between subsets. [file DataSheet_1.zip › Supplementary Figures/Supplementary Figure 1.tif]

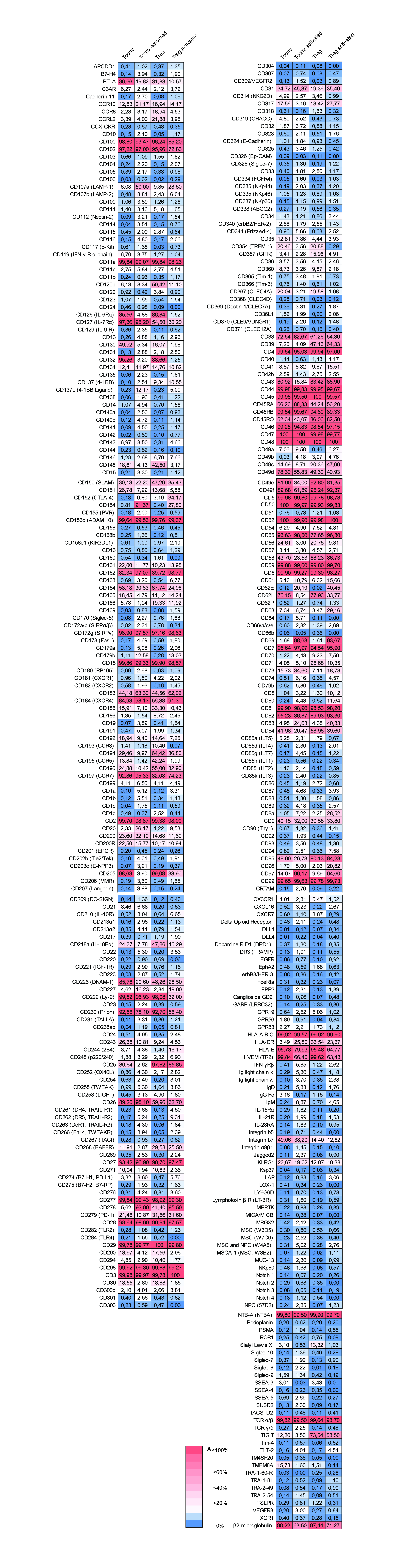

Supplement: Supplementary Figure 1 — Identification of Treg and Tconv cells. A. Representative dot plots for identification of Treg cells based on the expression of CD25 and FOXP3 or CD25 and CD127. B. Correlation between the frequencies of Treg cell subsets obtained by two different identification methods. Each symbol represents one donor (n=33). C. Representative dot plots and the gating strategy used to evaluate the expression of surface markers on Treg and Tconv cells (Tregs were identified as CD25+CD127lo/− cells among a CD4+CD14− population), followed by assessment of the expression of distinct markers between subsets. [file DataSheet_1.zip › Supplementary Figures/Supplementary Figure 2.tif]

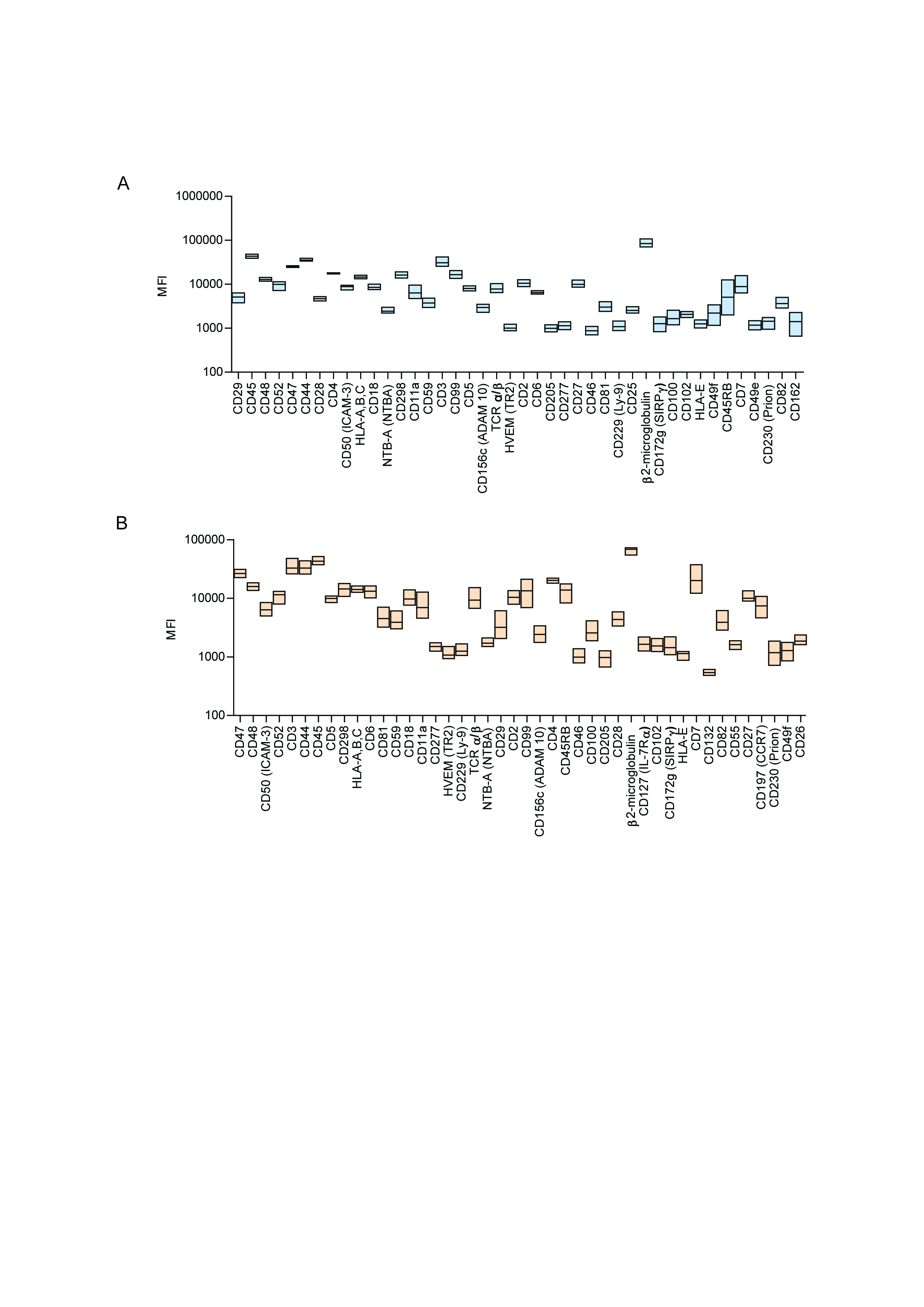

Supplement: Supplementary Figure 1 — Identification of Treg and Tconv cells. A. Representative dot plots for identification of Treg cells based on the expression of CD25 and FOXP3 or CD25 and CD127. B. Correlation between the frequencies of Treg cell subsets obtained by two different identification methods. Each symbol represents one donor (n=33). C. Representative dot plots and the gating strategy used to evaluate the expression of surface markers on Treg and Tconv cells (Tregs were identified as CD25+CD127lo/− cells among a CD4+CD14− population), followed by assessment of the expression of distinct markers between subsets. [file DataSheet_1.zip › Supplementary Figures/Supplementary Figure 3.tif]

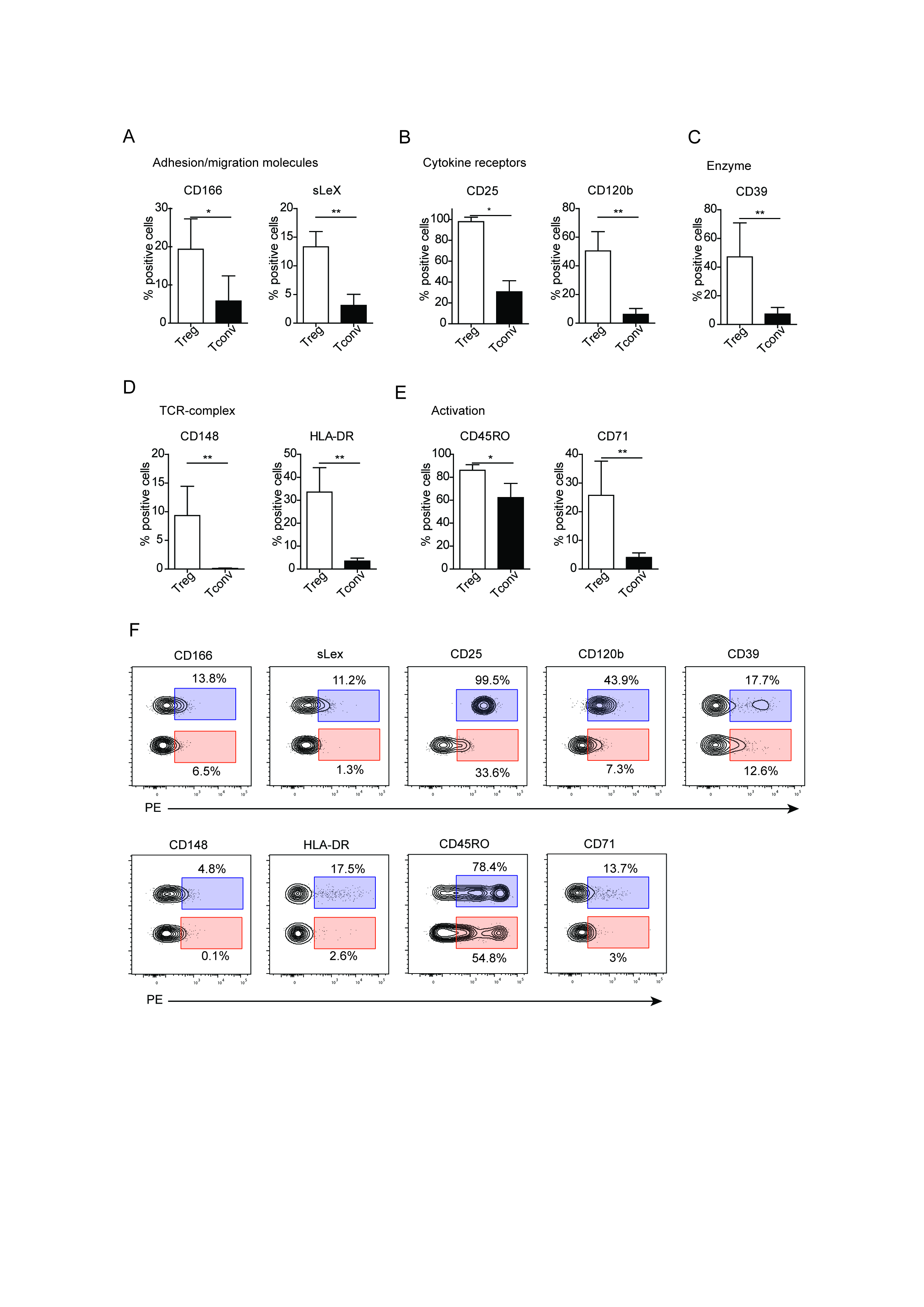

Supplement: Supplementary Figure 1 — Identification of Treg and Tconv cells. A. Representative dot plots for identification of Treg cells based on the expression of CD25 and FOXP3 or CD25 and CD127. B. Correlation between the frequencies of Treg cell subsets obtained by two different identification methods. Each symbol represents one donor (n=33). C. Representative dot plots and the gating strategy used to evaluate the expression of surface markers on Treg and Tconv cells (Tregs were identified as CD25+CD127lo/− cells among a CD4+CD14− population), followed by assessment of the expression of distinct markers between subsets. [file DataSheet_1.zip › Supplementary Figures/Supplementary Figure 4.tif]

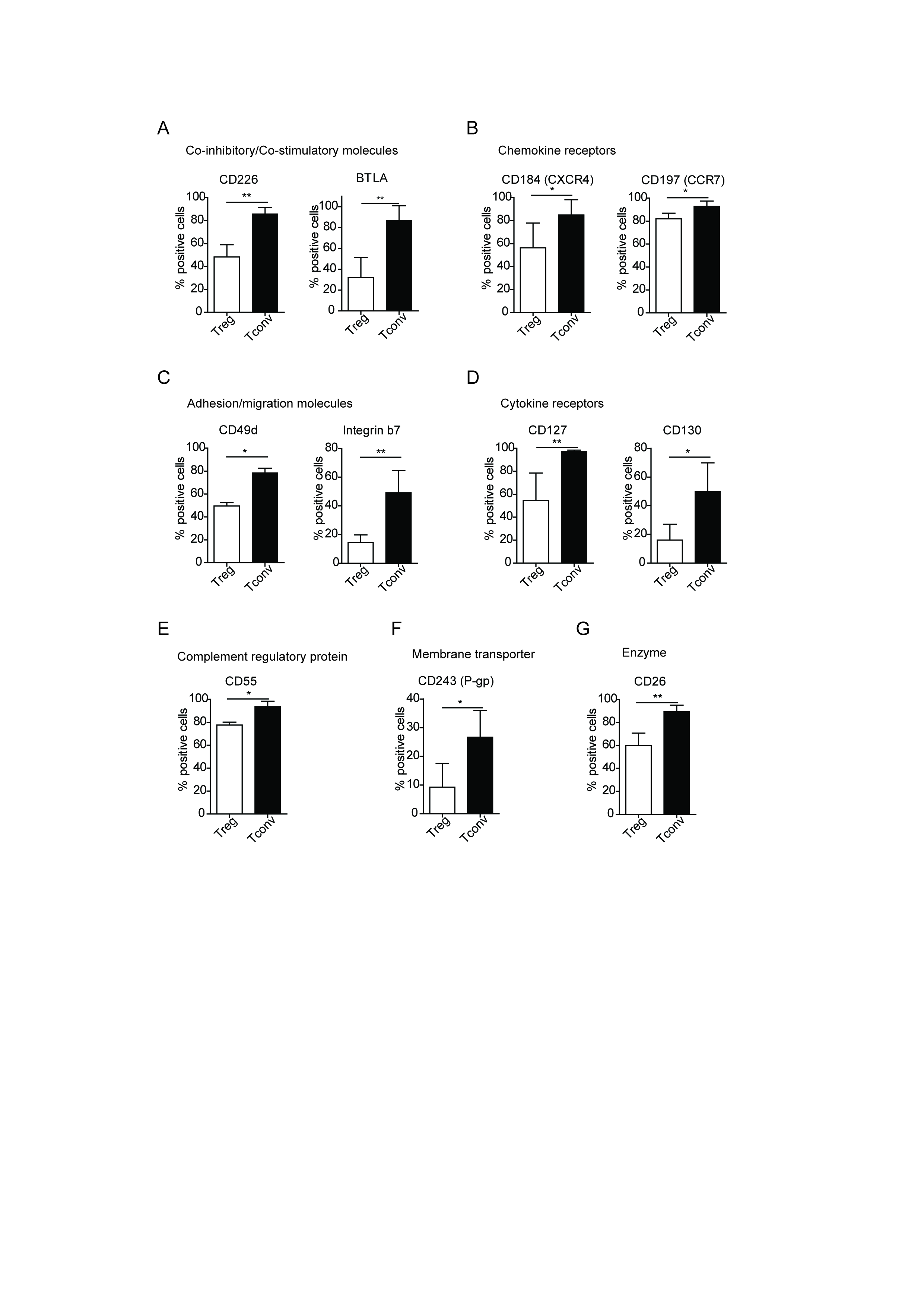

Supplement: Supplementary Figure 1 — Identification of Treg and Tconv cells. A. Representative dot plots for identification of Treg cells based on the expression of CD25 and FOXP3 or CD25 and CD127. B. Correlation between the frequencies of Treg cell subsets obtained by two different identification methods. Each symbol represents one donor (n=33). C. Representative dot plots and the gating strategy used to evaluate the expression of surface markers on Treg and Tconv cells (Tregs were identified as CD25+CD127lo/− cells among a CD4+CD14− population), followed by assessment of the expression of distinct markers between subsets. [file DataSheet_1.zip › Supplementary Figures/Supplementary Figure 5.tif]

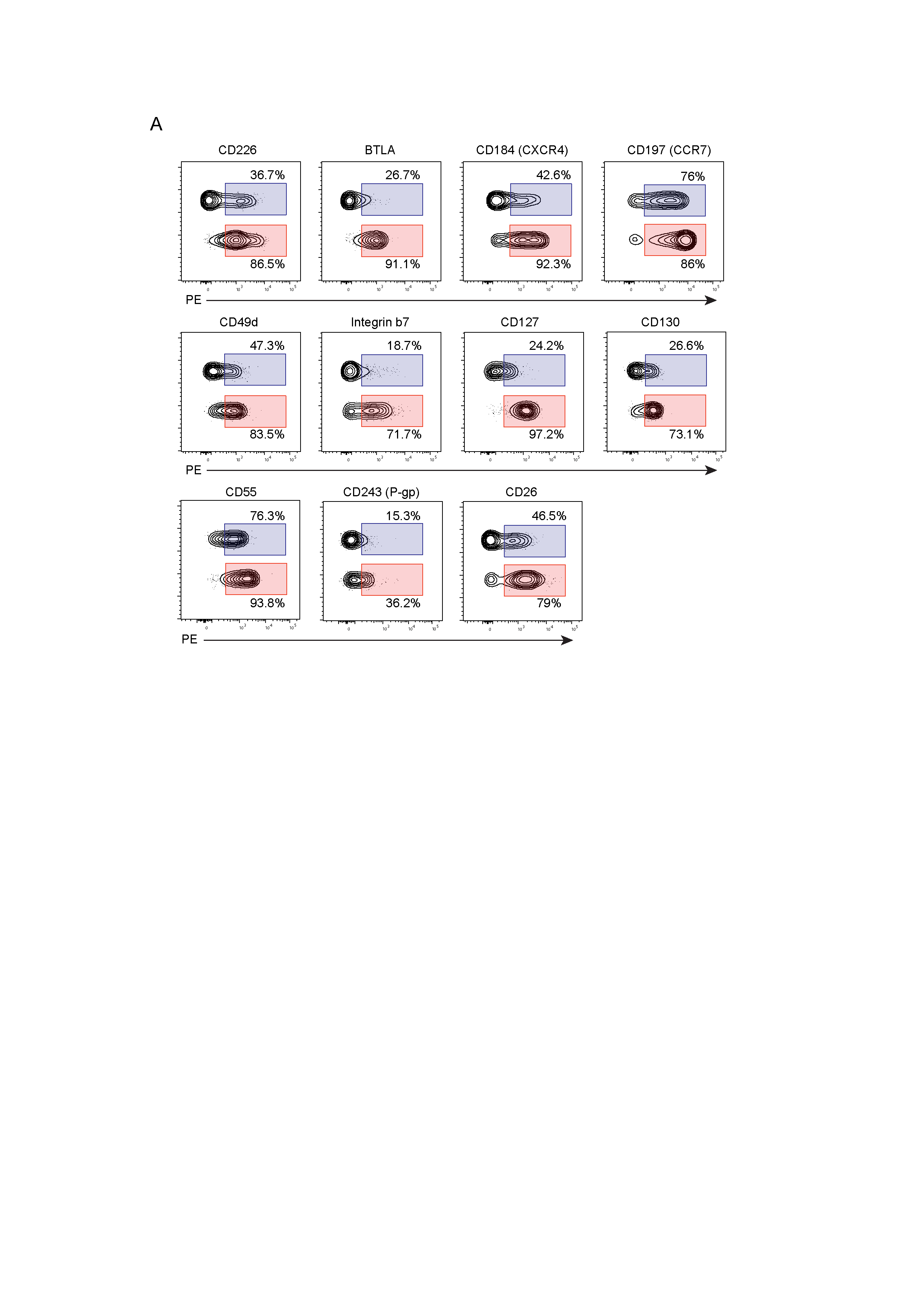

Supplement: Supplementary Figure 1 — Identification of Treg and Tconv cells. A. Representative dot plots for identification of Treg cells based on the expression of CD25 and FOXP3 or CD25 and CD127. B. Correlation between the frequencies of Treg cell subsets obtained by two different identification methods. Each symbol represents one donor (n=33). C. Representative dot plots and the gating strategy used to evaluate the expression of surface markers on Treg and Tconv cells (Tregs were identified as CD25+CD127lo/− cells among a CD4+CD14− population), followed by assessment of the expression of distinct markers between subsets. [file DataSheet_1.zip › Supplementary Figures/Supplementary Figure 6.tif]

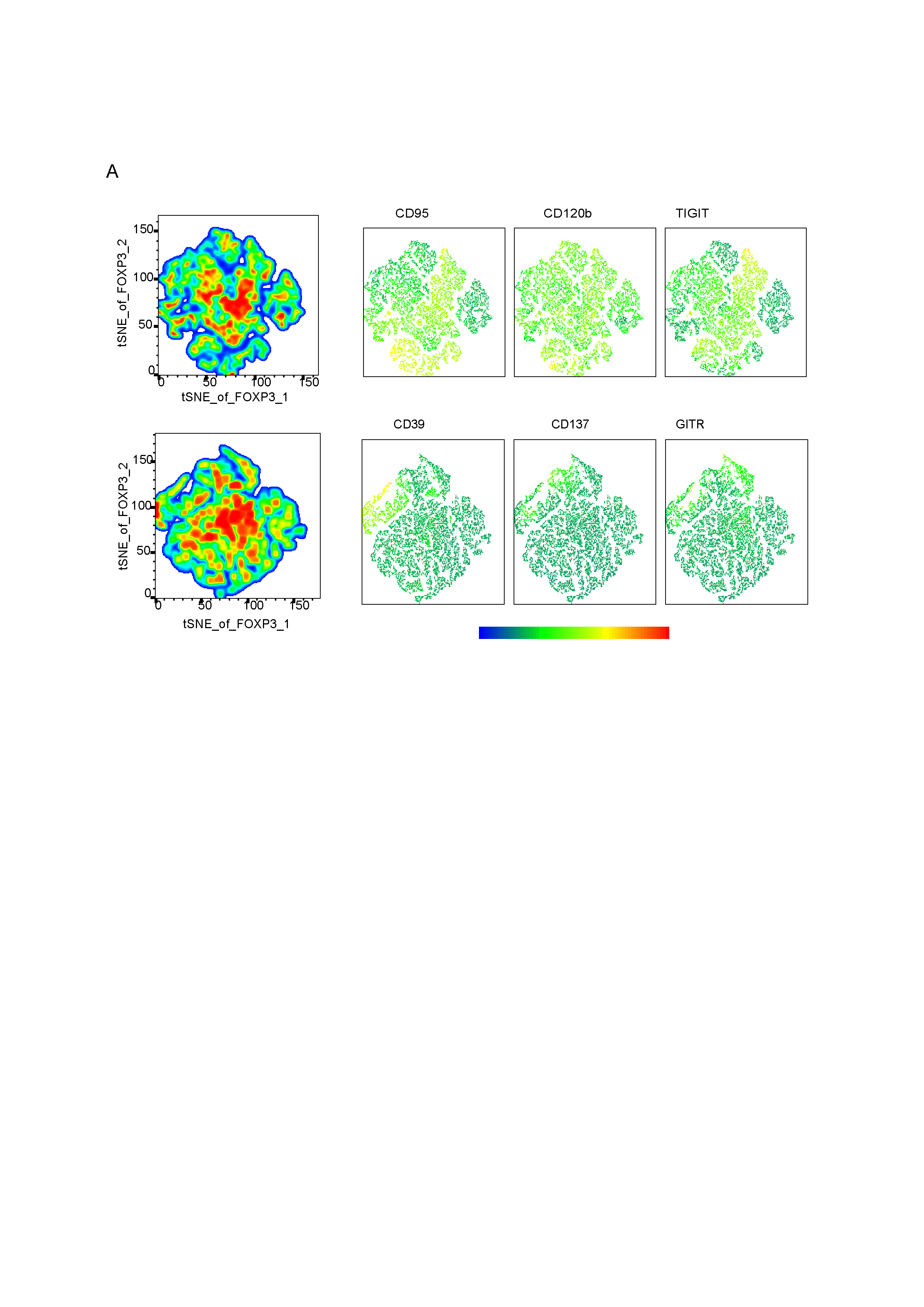

Supplement: Supplementary Figure 1 — Identification of Treg and Tconv cells. A. Representative dot plots for identification of Treg cells based on the expression of CD25 and FOXP3 or CD25 and CD127. B. Correlation between the frequencies of Treg cell subsets obtained by two different identification methods. Each symbol represents one donor (n=33). C. Representative dot plots and the gating strategy used to evaluate the expression of surface markers on Treg and Tconv cells (Tregs were identified as CD25+CD127lo/− cells among a CD4+CD14− population), followed by assessment of the expression of distinct markers between subsets. [file DataSheet_1.zip › Supplementary Figures/Supplementary Figure 7.tif]
